# [Retracted] MicroRNA‑326 inhibits cell proliferation and invasion, activating apoptosis in hepatocellular carcinoma by directly targeting LIM and SH3 protein 1

**DOI:** 10.3892/or.2023.8524

**Published:** 2023-03-13

**Authors:** Shiping Hu, Yun Ran, Wenlin Chen, Yuncheng Zhang, Yongjian Xu

Oncol Rep 38: 1569–1578, 2017; DOI: 10.3892/or.2017.5810

Following the publication of the above paper, it was drawn to the Editors' attention by a concerned reader that various panels showing data from flow cytometric experiments in Figs. 2E, 5E and 6E, and the cell migration and invasion assay data shown in Fig. 2D and 6D, were strikingly similar to data appearing in different form in other articles by different authors. Owing to the fact that the contentious data in the above article were already under consideration for publication, or had already been published, elsewhere when it was submitted to *Oncology Reports*, the Editor has decided that this paper should be retracted from the Journal. After having been in contact with the authors, they agreed with the decision to retract the paper. The Editor apologizes to the readership for any inconvenience caused.

